# Independent predictors of acute kidney injury in patients with acute coronary syndrome after percutaneous coronary intervention

**DOI:** 10.1371/journal.pone.0247304

**Published:** 2021-03-04

**Authors:** Aisulu Zhunuspekova, Jamilya Mansurova, Lyudmila Karazhanova

**Affiliations:** 1 Department of Cardiology and Interventional Arrhythmology, Semey Medical University, Semey, Kazakhstan; 2 Head Office, Semey Medical University, Semey, Kazakhstan; Thomas Jefferson University, UNITED STATES

## Abstract

**Introduction:**

High residual platelet reactivity (RPR) in patients after percutaneous coronary intervention (PCI) receiving antiplatelet agents has been associated with a high risk of developing acute kidney injury (AKI).

**Study aim:**

This study aimed at identification of independent prognostic predictors of AKI risk in patients with acute coronary syndrome (ACS) after PCI.

**Study design, setting and patients:**

This was a prospective single-center clinical trial that included 155 patients (n = 119 without AKI, n = 36 with AKI, mean age 64.0±10.6 years, of whom 74.2% were males), who underwent PCI with stenting. We prospectively evaluated RPR using optical aggregometry. Development of AKI was the primary endpoint.

**Results:**

Acute renal dysfunction was observed in 36 patients (23.2%) after PCI, the risk factors of which according to univariate regression analysis were: age (p = 0.040), low diastolic blood pressure (DBP) (p = 0.001), having severe heart failure (HF) according to Killip (p<0.001), low level of hemoglobin (p = 0.026) and erythrocytes (p = 0.005), increased creatinine (p<0.001), low baseline glomerular filtration rate (GFR) (p<0.001), low left ventricular ejection fraction (LV EF) (p = 0.003), high residual platelet reactivity (RPR) (p<0.001) and platelet aggregation area under the curve (AUC) with 10 μg/mL ADP (p<0.001), as well as dose of X-ray contrast medium (XCM) (p = 0.008). As a result of multivariate regression analysis the following independent predictors of AKI were established with the inclusion of the above factors: baseline creatinine level [OR 1.033 at 95% CI from 1.017 to 1.049; p<0.001], RPR with 10 μg/mL ADP [OR 1.060 at 95% CI from 1.027 to 1.094; p = 0.001], dose of an XCM [ОR 1.005 at 95% CI from 1.001 to 1.008; р = 0.014], diastolic blood pressure (DBP) [OR 0.926 at 95% CI from 0.888 to 0.965; p<0.001].

**Conclusion:**

ADP-induced high residual platelet reactivity, baseline creatinine level, X-ray contrast medium, low diastolic blood pressure were independent predictors of AKI in patients with ACS after PCI.

## Introduction

Cardiovascular disease is the leading cause of death worldwide [1]. Acute coronary syndrome (ACS) is a clinical manifestation of exacerbation of coronary heart disease.

Numerous studies have shown that in patients with ACS, acute renal dysfunction occurs in one of five cases and is one of the leading predictors of cardiovascular complications and mortality in the hospital period and in the long term [2–5].

When performing reperfusion therapy in patients with ACS, mechanical revascularization is the strategy of choice compared to the pharmacological reperfusion method [6]. Dual antiplatelet therapy (DAPT) both before and after PCI is one of the fundamental aspects of prevention of ischemic complications in patients with ACS [7]. The prognostic value of platelet hyperactivity in the development of AKI in this category of patients is little studied. This procedure requires the use of iodine-containing XCM which have a toxic effect on the renal tubule epithelium and changes in renal hemodynamics. A moderate decrease in kidney function is potentially important in critical conditions and in studies of contrast-induced nephropathy (CIN) [8].

The mechanism of development of AKI of ACS has not yet been fully studied, and the measures for its prevention are not unified. Therefore, prediction of acute kidney injury is critical.

To improve outcomes in patients with ACS after PCI performed with XCM, early identification of patients at high risk for acute renal impairment is necessary. The aim of this study was to identify independent predictors of AKI in patients with ACS after myocardial revascularization.

## Materials and methods

### Study design, setting and patients

The present prospective study included 155 patients with ACS, who were hospitalized in the cardiac surgery department of the University Hospital of Semey Medical University (Kazakhstan) from 2018 to 2019. All patients had undergone PCI and were prospectively evaluated for PRP using optical aggregometry induced by adenosine diphosphate (ADP) and epinephrine. Inclusion criteria: patients with ST-elevation acute coronary syndrome (STE-ACS) and non ST-elevation acute coronary syndrome (NSTE-ACS) according to coronaroangiography data, coronary artery disease requiring PCI with stenting; taking DAPT with a P_2_Y_12_ receptor inhibitor and acetylsalicylic acid (ASA).

Exclusion criteria: terminal renal failure in program hemodialysis patients, incidence of oncological diseases, intolerance to antiplatelet agents, refusal to participate in the study.

The study design was approved by the local ethics committee and followed the principles of Helsinki Declaration. Study design was аpproved by Local Ethical Committee of "Medical University Semey" Non-Commercial Joint-Stock Company Protocol 9 dated September 13, 2017. All patients gave written informed consent to participate in the study.

### Intervention

All patients received 325 mg of aspirin and a loading dose of 600 mg of clopidogrel or ticagrelor 180 mg, followed by a maintenance dose of 325 mg daily of aspirin and 75 mg daily of clopidogrel or ticagrelor 90 mg daily.

### Key outcome indicators

Acute kidney injury was the primary endpoint, which was defined according to the recommendation of KDIGO (2012) as an increase in serum creatinine (Cr) level ≥26.5 μmol/L within 48 hours after PCI compared to the initial indicator [9–12]. To determine the filtration function of the kidneys, as the most optimal, the glomerular filtration rate was calculated using the Cockcroft-Gault formula [13].

### Testing platelet aggregation activity

Aggregatometry is based on the ability of platelets to activate and form cell aggregates under the influence of inductors. During the analysis, changes in the light transmission of platelet-rich plasma are recorded as a result of the interaction of platelets through the IIb/IIIa glycoprotein receptors in response to stimulation by the inducer. This principle is used in the operation of an optical aggregometer. When assessing platelet aggregation according to G. Born and J. O’Brien, platelets are activated at 37^0^С by adding one of the agonists [14, 15]. After that, platelet aggregation occurs and transparency in the blood sample increases, which leads to an increase in the aggregation curve. Blood sampling was performed 12–48 hours after PCI in the morning on an empty stomach from the cubital vein into vacuum tubes with 3.8% sodium citrate (in a ratio of 9:1) with a short-term application of a tourniquet with a needle diameter of 0.8 mm. Blood samples were delivered to the laboratory within 20–30 minutes. To obtain platelet-rich plasma, a test tube with blood was rotated in an OPn-3.02 "Dastan" centrifuge (Kyrgyzstan) at 1000 rpm. within 10 minutes. The platelet-rich plasma tube was re-centrifuged at 3000 rpm for 15 minutes. The antithrombotic efficacy of antiplatelet agents was assessed on an AggRAM Helena Biosciences Europe aggregometer using adenosine-5’-diphosphate (ADP) and epinephrine from Tekhnologiya-Standard (Russia) at a dilution of 10 μg/mL and 2.5 μg/mL, respectively. The aggregation registration time was 10 minutes. The results of the study of platelet function were assessed by the maximum percentage of aggregation (max %) in response to stimulation with the inductor and the AUC. Platelet-poor plasma was taken as 100%, platelet-rich plasma as 0%. dx.doi.org/10.17504/protocols.io.bk6skzee

### Coronaroangiography

Coronary angiography was performed on a Siemens Artis zee angiographic unit using the standard M. Judkins technique with transfemoral or radiation access, subsequent digital x-ray processing, and DVD recording. According to the Seldinger method, a puncture of the femoral or radial artery was performed under a local anesthesia, followed by the installation of a hemostatic introducer sheath. To visualize coronary arteries, a low osmolarity XCM–Iohexol–was used. A hemodynamically significant criterion was considered to be a narrowing of the lumen of the vessel over 70% in diameter. Based on the results of the CAG and technical capabilities, simultaneous PCI was performed. X-ray data of all patients were recorded in the Protocol of coronary angiography.

### Statistical analysis

Statistical data processing was carried out using the SPSS 20.0 program. To check the correspondence of the real distribution of variables to the normal one, the Kolmagorov-Smirnov test was used. Quantitative variables with normal distribution were presented as mean values and their standard deviations (M±SD), min and max values; for comparison, the Student’s t-test was used for independent samples. In case of an abnormal distribution, quantitative variables are presented in the form of the median and interquartile range (Me(IQR)), min and max values; for their comparison, the Mann-Whitney U-test was used. Dichotomous signs are presented in the form of shares (absolute number of patients (%)), to identify the relationship between nominal variables χ^2^ Pearson, correction for continuity, Fisher’s test. Using binary logistic regression (univariate and multivariate), independent predictors of AKI and odds ratio (OR) were established at 95% confidence intervals (CI) for each factor. Using the ROC analysis, the predictive value of quantitative variables was estimated, their critical values were established, at which the event will occur with a probability of 0.5. Differences between the compared variables were considered significant at p <0.05.

## Results

The present study included 155 patients whose average age was 64.0±10.6 years old with a minimum and maximum being 29 and 87 respectively.

Among them, there were 115(74.2%) men (average age 62.2±10.6 years) and 40(25.8%) women (average age 69.3±8.9 years). Men were significantly younger than women (p<0.001). There were no statistically significant differences in the development of acute renal dysfunction in the patients with STE-ACS and NSTE-ACS (p = 0.715), therefore the calculation was performed for the entire group of patients. Comparative clinical and laboratory characteristics of patients in groups with AKI (n = 36; 23.2%) and without AKI (n = 119; 76.8%) are presented in Table 1.

**Table 1:** General clinical characteristics of patients depending on the presence of acute kidney injury.

Indicator	All patients (n = 155)	Patients without AKI (n = 119)	Patients with AKI (n = 36)	p-value
Age, years	64.0±10.6	63.0±10.5	67.2±10.5	0.038*
	29–87	29–87	38–87	
Male, n(%)	115(74.2)	87(73.1)	28(77.8)	0.731^с^
BMI, kg/m^2^	27.0(6.7)	27.6(7.0)	26.7(4.9)	0.190**
	18–80	18–80	20.2–41.4	
Pre-existing arterial hypertension, n(%)	152(98.1)	116(97.5)	36(100)	1.000^b^
Diabetes mellitus, n(%)	31(20)	25(21.0)	6(16.7)	0.739^с^
Smoking, n(%)	56(36.1)	45(37.8)	11(30.6)	0.427^a^
STE-ACS, n(%)	95(61.3)	72(60.5)	23(63.9)	0.715^a^
ABP systolic, mm Hg	130(20)	130(20)	120(38)	0.069**
	40–210	40–210	40–180	
ABP diastolic, mm Hg	80(10)	80(10)	80(25)	0.001**
	0–100	40–100	0–90	
Clopidogrel	91(58.7)	73(61.3)	18(50)	0.226^a^
Ticagrelor	64(41.3)	46(38.7)	18(50)	0,226^a^
**Heart Failure (Killip)**				
I	80(51.6)	64(53.8)	16(44.4)	0.326^a^
II	56(36.1)	50(42)	6(16.7)	0.006^a^
III	1(0.6)	1(0.8)	-	1.000^b^
IV	18(11.6)	4(3.4)	14(38.9)	<0.001^b^
LV EF, %	51(9)	51(8)	49,5(12)	0.010**
	23–66	29–66	23–58	
IACBS, n(%)	3(1.9)	2(1.7)	1(2.8)	0.550^b^
**Laboratory indicators**				
Hemoglobin, g/L	144(23)	145(20)	136,5(33)	0.033**
	64–180	64–180	80–174	
Erythrocytes	4,6(0.7)	4.6(0.6)	4.3(1.0)	0.003**
	2.5–5.7	2.5–5.7	2.8–5.5	
Cholesterol, mmol/L	4.6(1.7)	4.7(1.7)	4,2(2.0)	0.238**
	2.3–9.0	2.5–9.0	2.3–8.1	
Triglycerides, mmol/L	1.5(1.0)	1.6(1.0)	1.5(1.0)	0.339**
	0.5–5.0	0.6–5.0	0.5–4.1	
Glucose, mmol/L	6.6(3.0)	6.5(2.9)	6.9(3.7)	0.936**
	2.9–20.7	4.0–20.7	2.9–12.5	
Creatinine baseline, μmol/L	101(45)	97(38)	128.7(82)	<0.001**
	37–701	37–218	53–701	
GFR baseline	58.0±23.1	63.4±21.0	40.3±20.8	<0.001*
	8–114	21–114	8–111	
Calcium, mmol/L	1.2(0.1)	1.2(0.1)	1.1(0.1)	<0.001**
	0.9–2.8	0.9–2.8	1.0–2.4	
**Residual Platelet Reactivity against the background of DAPT**				
RPR with 10 μg/mL ADP, %	46,8(33.8)	41.5(30.9)	61.7(23.7)	<0.001**
	10.5–83.4	10.5–83.4	29.2–82.9	
AUC with 10 μg/mL ADP	37.5(36.0)	28.8(34.6)	51.1(24.0)	<0.001**
	0.9–77.4	0.9–77.4	15.2–76.2	
RPR with 2,5 μg/mL epinephrine, %	34.0±19.4	32.9±18.3	37.6±22.4	0.212**
	0.4–84.7	0.4–79.8	0.4–84.7	
AUC with 2,5 μg/mL epinephrine	23.6±16.0	22.8±15.1	26.3±18.5	0.251**
	0–68.2	0–64.8	0–68.2	
**X-ray contrast medium**				
Contrast, mL	200(100)	200(100)	300(175)	0.007**
	100–800	100–800	100–800	

*Note*.

*—parametric criteria—student t-test, M±SD (mean ± standard deviation); min and max values

** nonparametric test—Mann-Whitney U-test, Me(IQR) (median(interquartile range)); min and max values; nominal variables (absolute number of patients (%))

^a^– *χ*^2^ Pearson

^b^—Fisher test

^c^—correction for continuity; AKI—acute renal injury; BMI—body mass index; STE-ACS—acute coronary syndrome with ST elevation; BP–blood pressure; IACBS- intra aortic counterpulsation balloon support; LV EF—left ventricular ejection fraction; GFR–glomerular filtrate rate; RPR—residual platelet reactivity; AUC—aggregation area under the platelets curve; ADP—adenosinediphosphate.

The incidence of AKI in the study population was 36(23.2%) cases. Table 2 shows the results of a single-factor regression analysis, which identified statistically significant risk factors for AKI against the background of dual antiplatelet therapy.

**Table 2:** Risk factors for AKI in patients with ACS on the background of DAT.

Indicator	OR	95% CI		p-value
		Lower	Upper	
Age	1.041	1.002	1.081	0.040
DBP	0.948	0.919	0.978	0.001
Degree of HF (Killip)	2.190	1.496	3.206	<0.001
Hemoglobin	0.981	0.965	0.998	0.026
Erythrocytes	0.390	0.203	0.747	0.005
Creatinine	1.026	1.013	1.038	<0.001
Baseline GFR	0.940	0.917	0.965	<0.001
LVEF	0.929	0.885	0.975	0.003
RPR with 10 μg/mL ADP	1.046	1.023	1.070	<0.001
AUC with 10 μg/mL ADP	1.041	1.020	1.063	<0.001
XCM	1.004	1.001	1.007	0.008

*Note*. OR–odds ratio, CI—confidence interval, DBP–diastolic blood pressure, HF—heart failure, GFR–glomerular filtration rate, LVEF—left ventricular ejection fraction, RPR—residual platelet reactivity; AUC—area under the platelet aggregation curve, ADP–adenosine diphosphate, XCM—X-ray-contrast medium.

When the above variables were included in the multivariate regression analysis, the following independent predictors of AKI were statistically significant: creatinine [OR 1.033 at 95% CI from 1.017 to 1.049; p<0.001], RPR with 10 μg/mL ADP [OR 1.060 at 95% CI from 1.027 to 1.094; p = 0.001], X-ray-contrast medium dose [OR 1.005 at 95% CI from 1,001 to 1.008; p = 0.014], diastolic blood pressure (DBP) [OR 0.926 at 95% CI 0.888 to 0.965; p<0.001]. The accuracy of the forecast when including the established independent predictors of AKI was 87.1%, sensitivity– 58.3% and specificity– 95.8%.

**Fig 1:**
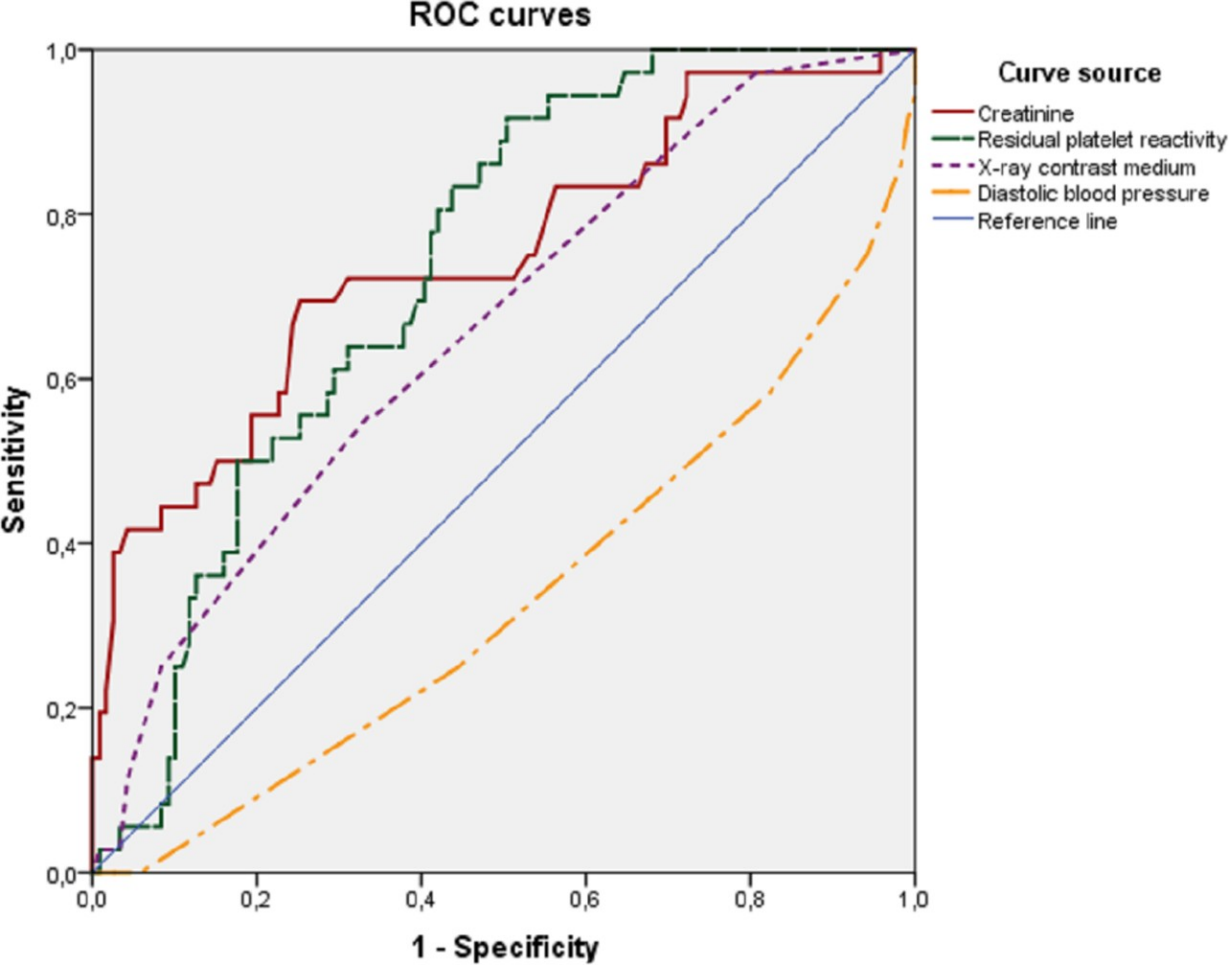
ROC curves of independent variables. ROC-curves of independent predictors of AKI in patients with ACS after PCI (creatinine, residual platelet reactivity, X-ray contrast medium, diastolic blood pressure), which demonstrate the relationship between the sensitivity and specificity of tests.

To determine the optimal cut—off points for quantitative variables, a ROC analysis was performed with the construction of ROC curves (Fig 1).

The areas under the ROC curves for independent predictors of AKI development are shown in Table 3.

**Table 3:** Areas under the ROC curves of independent predictors of AKI development.

Indicator	Area	95% CI		p-value
		Lower	Upper	
Creatinine	0.748	0.650	0.846	<0.001
RPR with 10 μg/mL ADP	0.733	0.652	0.814	<0.001
XCM	0.659	0.560	0.758	0.004
Diastolic blood pressure	0.333	0.229	0.438	0.002

*Note*. DI–confidence interval, RPR—residual platelet reactivity; ADP—adenosinediphosphate, XCM—X-ray-contrast medium, BP–blood pressure.

In the study population, the optimal cut—off points for variables with the highest sensitivity and specificity were: creatinine level 119 μmol/L, RPR value 45%, X-ray contrast medium dose 275 ml, diastolic blood pressure 70 mm Hg.

The following multivariate mathematical model was obtained to calculate the probability of the onset of AKI in miltifactorial logistic regression:

$$P=1/1+е{‐}^{\left(0,032*С+0,058*RPR+0,005*XCM–0,077*DBP–3,342\right)},$$

where P*—*likelihood of AKI, *e*—the base of the natural logarithm, approximately equal to 2.718; C—creatinine; RPR—residual platelet reactivity induced by ADP; XCM—X-ray contrast medium; DBP—diastolic blood pressure. If the value of P is greater than 0.5, then the onset of the AKI is expected, otherwise the event will not occur.

**Fig 2:**
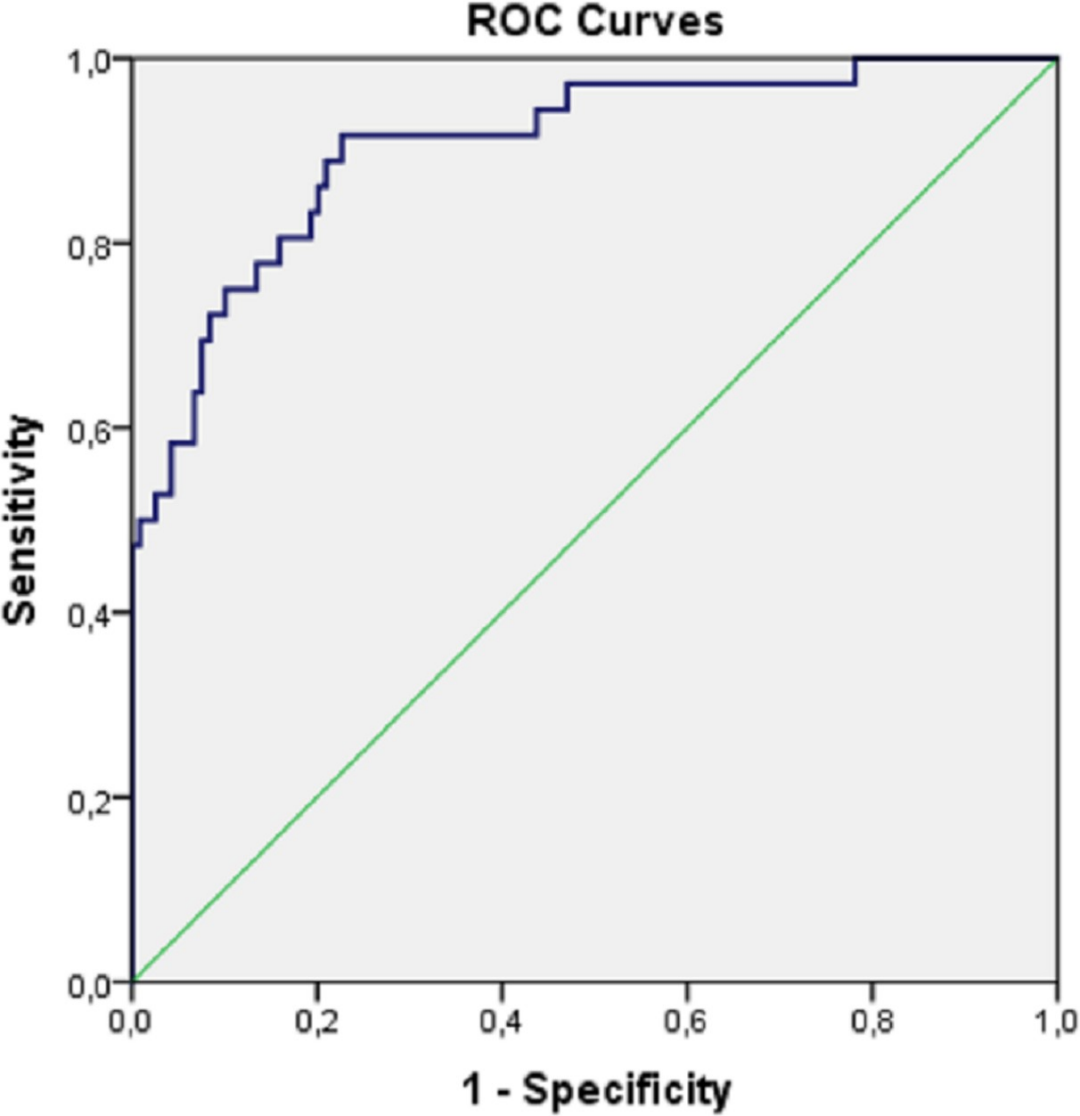
ROC curve of predictive ability of a regression model of AKI prognosis. The area under the ROC curve for the predicted AKI probability values was 0.905 with 95% CI from 0.847 to 0.964 (p<0.001), which corresponds to a very good predictive power of the model.

To assess the quality of the obtained predictive model, we used ROC analysis with the inclusion of predicted values of the probability of AKI development (Fig 2).

Given the presence of a multi-factor predictive model, the optimal value of one factor depends on the indicators of other factors that affect the development of the event.

## Discussion

Acute renal dysfunction is a common complication that can develop in patients with ACS and reach the rate of 30% [16–19]. In our study population, acute renal dysfunction developed in 23.2% of patients.

The key result of this study was the identification of independent predictors for the development of acute renal dysfunction in patients with ACS after PCI with stenting. The baseline high creatinine level, high RPR (induced by 10 μg/mL ADP), the dose of XCM, low diastolic blood pressure were the main factors for the development of AKI against the background of DAPT, including ASA and clopidogrel/ticagrelor. In binary regression analysis, a multivariate mathematical model of risk was obtained that allows calculating the probability of AKI in patients with ACS after PCI with stenting. The prognostic model we obtained included such factors as ORT, DBP level that can change during therapy, i.e. by optimizing treatment, the likelihood of AKI can be reduced, thereby personalizing the therapy.

One of the important evaluation criteria in this study was platelet function testing by optical aggregometry induced by ADP. Currently, many methods are used to determine platelet aggregation activity. However, the effectiveness of antiplatelet therapy is not controlled, since there is still no standardized parameter by which platelet hyperactivity and the degree of its change against the background of antiplatelet therapy could be established [20, 21].

Several studies have established the dominant role of platelet function in the pathogenesis of ACS and the prognostic value of the high residual platelet reactivity associated with DAPT in the development of adverse cardiovascular events in patients undergoing PCI [22–24]. However, in this category of patients, the role of platelet hyperactivity in the development of AKI is not well understood. As far as we know, this is the first study to demonstrate that the development of AKI in patients with ACS after PCI is associated with high residual platelet reactivity and the area under the aggregation curve, which is recommended for scientific purposes.

Hakan Uçar et al. noted that the medium platelet volume (MPV) may serve as a marker of impaired renal function in patients with stable angina pectoris [25]. In a study by Gremmel T. et al., high residual platelet reactivity was found to be a predictor of the development of adverse clinical outcomes in patients with chronic kidney disease (CKD) with ACS after PCI [26]. Zdrojewski Z. and co-authors identified a direct correlation between the high aggregation activity of platelets, as well as an increase in their size with the progression of renal failure in patients with glomerulonephritis [27]. Also, C.M. Gasperetti et al. found that balloon angioplasty increases serotonin-induced platelet aggregation [28]. A systematic review and meta-analysis done by Jie Jiang et al. established the prognostic significance of the ratio of platelet to lymphocyte in the development of CIN, which develops after administration of XCM, and is one of the leading causes of acute renal failure at hospital stage [8, 29].

In this work, the role of platelets in the development of acute renal dysfunction was studied by determining the residual platelet reactivity by the ADP inducer. It was found that platelets are involved in the development of AKI since platelet hyperactivity has proven to be one of the important modifiable independent predictors of AKI in patients with ACS after PCI (p = 0.001).

One of the factors for the development of AKI with the introduction of XCM is the total amount of contrast used during the procedure. Irfan Sahin et al. found that an increase in the level of XCM (p = 0.01), age (p = 0.05) and the platelet distribution width (PDW) (p = 0.04) were independent predictors of the development of CIN in patients with ACS after coronarography and/or PCI [17]. According to the Cath-PCI register, a link was established between the volume of the contrast medium and the risk of renal injury. To achieve optimal intra-procedural results, the use of a minimum amount of a contrast dose should be emphasized in each case [30]. Our study has also identified the relationship between the volume of XCM used for patients with ACS and the risk of AKI (p = 0.014), optimal value of the cut-off point for the dose of XCM, at which sensitivity and specificity are maximum, was 275 mL (p = 0.008).

Numerous studies have shown that renal failure or chronic kidney disease (CKD) is an independent predictor of AKI in patients with ACS (p<0.001) [31]. The baseline serum creatinine level turned out to be the leading risk factor for AKI in the study population (p<0.001), creatinine levels 1.3 mg/dL (119 μmol/L) were the cut-off point (р<0,001). The ACEF risk model (2009), used to assess the risk of adverse cardiovascular complications in patients after surgical and percutaneous myocardial revascularization, includes factors such as age, creatinine, and left ventricular ejection fraction. At creatinine levels above 2 mg/dL (177 μmol/L), an increase in the frequency of the combined endpoint, including death from heart disease, acute myocardial infarction, or stroke, was observed [32, 33].

Patients with cardiogenic shock and initial CKD have a higher risk of developing AKI during PCI [30]. Study by Tarun Jain et al. demonstrated the relationship between the risk of developing CIN and cardiogenic shock. It was shown that patients with a cardiogenic shock at the time of PCI had significantly greater risks of developing CIN (p = 0.003) [34]. In our study, AKI was significantly more likely to develop in patients with cardiogenic shock (Killip IV) than in patients with normal blood pressure (38.9% vs 3.4%, p<0.001). Low diastolic blood pressure was an independent predictor of AKI (p<0.001). Thus, the presence of any hypotension during PCI can be considered one of the most reliable predictors of AKI.

There is a Cleveland score (2005) risk stratification model for assessing AKI after heart surgery that includes variables such as gender, chronic heart failure, left ventricular ejection fraction, preoperative use of an intra-aortic balloon pump, chronic obstructive pulmonary disease, diabetes mellitus, heart surgery, type of surgery, emergency surgery, baseline creatinine level [35]. The above factors are mainly nominal non-modifiable in contrast to those obtained in our model. When assessing the risk of developing CIN after PCI, the Mehran scale is used, which includes predictors such as hypotension, age, congestive heart failure, anemia, diabetes mellitus, creatinine level, XCM volume, and the use of an intra-aortic balloon pump [36]. The risk scales do not take into account laboratory indicators, such as residual platelet reactivity and platelet aggregation area under the curve, which may vary depending on the effectiveness of antiplatelet therapy, as well as patient adherence to treatment and lead to shifts in risk assessment.

Unfortunately, there is still no consensus on a personalized approach to prescribing DAPT. The results of large randomized trials, such as TRIGGER-PCI, GRAVITAS, ARCTIC, did not reveal the advantages of a personalized approach to prescribing DAPT to patients after planned PCI [37–39]. In contrast, a multicenter randomized trial, TROPICAL-ACS, established the benefit of personalized therapy using platelet function testing in clinical PCI results [40].

The results of our study indicate the need for a personalized approach in prescription of antiplatelet therapy based on platelet function testing, the correction of which can reduce the likelihood of AKI after PCI.

## Conclusion

This study has found that independent predictors of the development of acute renal dysfunction in patients with ACS after PCI with stenting against the background of DAPT are: baseline creatinine level (p<0.001), 10 μg/mL ADP-induced high RPR (p = 0.001), dose of XCM (p = 0.014), low DBP (p<0.001). RPR, dose of XCM, BP level are modifiable factors. By optimizing treatment aimed at correcting the obtained variables, it is possible to reduce the likelihood of AKI, thereby personalizing the therapy.

Thus, high RPR in patients with ACS 12–48 hours after PCI, detected by optical aggregometry, indicates a high risk of AKI. Given the prognostic significance of platelet function testing, RPR should be determined in this category of patients to identify those at high risk of AKI.

A possible association between high platelet activity and progression of renal failure has not been adequately studied. The results of this work show the need for further prospective studies in this area as well as during other angiographic procedures.

## Supporting information

S1 Data (ZIP)
